# Genital self-sampling for HPV-based cervical cancer screening: a qualitative study of preferences and barriers in rural Ethiopia

**DOI:** 10.1186/s12889-019-7354-4

**Published:** 2019-07-31

**Authors:** Theresa Brandt, Solomon Berhe Wubneh, Simegnew Handebo, Getu Debalkie, Yohanes Ayanaw, Kassahun Alemu, Felix Jede, Magnus von Knebel Doeberitz, Hermann Bussmann

**Affiliations:** 1 Department of Applied Tumor Biology, Institute of Pathology, Heidelberg University Hospital, Im Neuenheimer Feld 224, 69120 Heidelberg, Germany; 2 Department of Gynaecology, School of Medicine, College of Medicine and Health Sciences, University of Gondar, Gondar, Ethiopia; 3 Institute of Public Health, College of Medicine and Health Sciences, University of Gondar, Gondar, Ethiopia; 4 Dabat Research Centre Health and Demographic Surveillance System, Institute of Public Health, College of Medicine and Health Sciences, University of Gondar, Gondar, Ethiopia

**Keywords:** Self-sampling, HPV self-sampling, HPV DNA testing, Evalyn® brush, Cervical cancer, Cervical cancer screening, Ethiopia, Gondar, Dabat HDSS, Focus group discussion self-sampling

## Abstract

**Background:**

In the context of WHO’s “task shifting” project and growing global consensus on primary HPV-based cervical cancer screening, self-sampling is a promising new tool to expand screening access, uptake and coverage for women worldwide. We aimed to explore perceptions and acceptability of HPV self-sampling-based cervical cancer screening among community members and health professionals in rural northwest Ethiopia and to identify preferences and socio-cultural barriers regarding self-sampling in order to design a suitable high-coverage screening intervention for a rural African setting.

**Methods:**

Four community-based focus group discussions (FGD) were conducted in the rural district of Dabat, Northwest Ethiopia, each comprising 8 to 14 female participants, counting a total of 41 participants. The groups were homogenously composed in terms of their socio-economic status in the community. They included health centre attendees, community members, nurses and health development army leaders (HDAL). Two qualitative data collection experts conducted the interviews in the local language, using a FGD guide with several thematic areas. All participants granted written informed consent prior to the conduct of the interviews. As a concrete example of an existing self-sampling approach for cervical cancer screening we used the Evalyn® Brush.

**Results:**

Emerging themes included (i) misconceptions and low awareness about cervical cancer among community residents and primary health care providers in rural northwest Ethiopia, (ii) stigmatization and social exclusion of affected women, (iii) delay in seeking of health care due to poor access and availability of services, and lacking of a concept of early cancer prevention, (iv) need of spousal permission, (v) fear of financial burden and (vi) fear of social marginalization. The self-sampling device was regarded to be acceptable and was judged to be easy to use for most women. The existing Ethiopian health care structure could facilitate a community approach.

**Conclusion:**

Home-based self-sampling for cervical cancer screening is a socially acceptable and feasible “task shifting” method that will increase cervical cancer screening access and coverage in the Ethiopian study community. Education, awareness creation, community mobilization and family inclusion are identified as key activities to promote, implement and facilitate “task shifting” approaches like self-sampling.

**Electronic supplementary material:**

The online version of this article (10.1186/s12889-019-7354-4) contains supplementary material, which is available to authorized users.

## Background

Cervical cancer remains one of the most common female cancers in low-resource settings [1] although highly preventable as shown in countries with functional screening programs [2–4]. Many obstacles hinder a successful fight against cancer in poor, rural and remote areas of the world including the unavailability or inefficacy of screening programs, underfunded and overburdened health services and high prevalence of HPV and HIV infections.

In 2006, the World Health Organization (WHO), in collaboration with the Office of the United States Global AIDS Coordinator (OGAC), has launched the WHO/OGAC Task Shifting Project. “Task shifting” is the delegation of responsibilities to less specialized health workers making more efficient use of limited medical personnel in LMIC (low and middle-income countries). It has been successfully implemented in HIV treatment and prevention, for instance qualified community workers delivering a wide range of HIV services, as a result freeing time of nurses and doctors [5–7].

Despite available vaccines against HPV infections, screening programs and preventive examinations will continue to play an important role in early cancer detection for all non-vaccinated, but also HPV vaccinated women all around the world. Regarding cervical cancer, HPV testing is considered the most effective screening approach combining high sensitivity with high negative predictive value [8–11]. The absence of a high risk (hr)HPV infection ensures low cancer risk for a considerable time period of at least 5–7 years as a persistent infection is needed for the development of cervical cancer [12–15].

Self-collection of genital samples for (hr)HPV DNA analysis are opening new ways for “task shifting” away from the few, centralized and remote health centres and clinics into the rural communities themselves. They have shown to perform equally well compared to provider-collected samples [16–20]. In Europe self-sampling is being increasingly tested in well-established programs as a means to engage screening defaulters e.g. by sending a self-sampling kit by post [21–27]. In a number of low resource settings high acceptance of self-sampling has been reported from studies done among women attending health facilities [28–33]. But only a few studies have also looked at women’s characteristics including socio-economic status, cultural background and prior knowledge and experience of cervical cancer screening [34–37]. The use of self-sampling as a home-based method to increase screening access and to relieve overburdened and understaffed health services will potentially face different challenges including knowledge, understanding, willingness, privacy, and socio-cultural issues.

Among Ethiopian women, cervical cancer is ranked second most common female cancer and second leading cause of cancer death [1, 38]. The vulnerable female population comprises 31.5 million women [39] making it a reproductive and maternal health issue of unique relevance. The Ethiopian government just recently launched a HPV vaccination program for 14-year-old girls and also started introducing visual inspection with acetic acid (VIA). However, over 80% of the population lives in rural areas of the country, leaving only 0.6% of all women with access to opportunistic rather than regular cervical cancer screening [39]. Hence, the impact of these recently established programs will take time. Home-based HPV-testing could therefore be a promising approach to initiate population-based screening programs. This is the first qualitative study to explore perceptions, acceptability, barriers and preferences of HPV self-sampling in a rural Ethiopian community naïve to screening practices in order to design a high-coverage cervical cancer screening intervention.

## Methods

### Study design

We undertook a qualitative study using community Focus Group Discussions (FGDs) and Key Informant Interviews (KIIs) to explore community members’ and health professionals’ knowledge about cervical cancer, as well as perceptions and acceptability of HPV self-sampling. In the health sector, FGDs are a popular method benefiting from particular group dynamics and interactions when generating data exploring knowledge, motives and experiences. However, when aiming to gather sensitive information KIIs can add qualitative depth as they are conducted in a one-on-one setting creating a more intimate and confidential environment in which potentially sensitive information can be shared without fear of repercussions [40, 41].

Through the FGDs and KIIs we aimed to identify specific preferences and socio-cultural barriers of the community concerning this cervical cancer screening approach to identify the best form for the function of HPV self-sampling and, thus, to design a suitable intervention for this rural African setting. Ethical approval was obtained from the Institutional Review Board (IRB) of the University of Gondar, as well as the IRB at Heidelberg University School of Medicine, Germany. All participants were provided with information about the study, were willing to participate and signed an Informed Consent Form in Amharic prior to the conduct of the interview.

### Setting

The study was conducted in the Dabat Health Centre in June 2017. The Dabat district in the Amharic region of Ethiopia contains 30 mainly rural communities (*kebele*) with each a population of approximately 5000 people. Families typically live as subsistence farmers on their homestead surrounded by small fields where they grow crops and herd their livestock. Each *kebele* has a health post staffed with community health extension workers (CHEW) responsible for health promotion and disease prevention in their communities. They closely work together with designated female residents of the *kebele*, the so called Health Development Army Leaders (HDAL), who have better insight, as well as influence on the households, health behaviour and habits of their *“gotes”* (smaller organised groups of the community).

### Data collection

All sexually active women between the ages of 20 and 65 years, permanently living in Dabat Town who were capable to understand the study procedures and were willing to voluntarily participate were eligible to enrol in the study. Saturation was reached after the conduct of four community FGDs with a total of 41 female participants, as well as four KIIs. FGD participants (see Additional file 1: Table S1) and key informants (see Additional file 2: Table S2) were identified opportunistically from the target community, as well as the local health service program. Two male qualitative data collection experts (researchers of health education and behavioural sciences and lecturers at the Public Health Institute at Gondar University, with a master’s degree in health education and behavioural science) lead and conducted the FGDs. The focus group (FG) topic guide was written in English and translated into the local language – *Amharic* – prior to the conduct of the FGDs. It was designed to gather information on knowledge and local perceptions of cervical cancer and women affected by it, knowledge about available preventive methods, possible facilitators and barriers for preventive screening, particularly in regard to the self-sampling device, the Evalyn® Brush and recommendations for implementation of a pilot screening study. Key topics were probed in each focus group (see Additional file 4).

The Evalyn® Brush was chosen as a concrete example of self-sampling technology because in previous studies it was well accepted by women; they found it comfortable and easy to use [17, 42–44]. Furthermore, it was shown to have a low inadequate specimen rate [17], as well as very good agreement with provider-taken specimens [17, 42, 44–46]. It can be stored dry at room temperature for transport [17, 42, 46] and can last up to 32 weeks without decreasing in diagnostic accuracy concerning HPV detection [47]. All of these factors are crucial when exploring the performance of self-sampling in a rural African health care setting.

Each FGD (and KII) was conducted in Amharic and lasted 60–90 min. They were audio-recorded after having obtained written informed consent and notes were taken both on verbal and non-verbal communication.

In order to avoid intimidation by hierarchical structures and to allow free and open discussion among the participants, the groups were formed homogenously in terms of their socio-economic status in the community. FGD participants sat in a circle and introduced themselves anonymously using code names which were used for reference during the discussions, as well as in all transcripts and notes associated. However, there is no absolute certainty that all participants were complete strangers to each other. The two facilitators enabled a free and open atmosphere for discussion and encouraged the women to actively participate at all times throughout the discussion.

A male senior resident of the Obstetrics and Gynaecology department at Gondar University Hospital assisted in the conduct of the interviews and helped with emerging medical questions. He introduced and explained the self-sampling device, as well as the exact screening procedure to the participants. In detail, a demonstration video on how to use the Evalyn® Brush (translated into Amharic) was shown to the participants. Furthermore, an exemplary brush was demonstrated and the self-collection procedure was explained a second time. Then the brushes were passed around to allow each woman to get a physical impression of the device.

### Data analysis

The two qualitative data collection experts transcribed all audio-recordings and field notes into Amharic and translated them into English. The datasets were analysed through thematic analysis.

Themes and sub-categories were induced from the transcripts through repeated reading. Majoritarian key messages, attitudes, perceptions and thoughts with particular emerging concepts were identified and coded. Responses from individual participants were put into sub-categories and broken down into units of sentences and paragraphs that represented particular thoughts of participants. Repeated ideas were categorized into broader themes (groups of repeated ideas) in order to create a theoretical narrative. Once themes and sub-categories were established, the transcripts were re-read to ensure they appropriately reflected the content of the data. Themes were then compared between FGD groups to identify similarities and differences. The qualitative data serves as prior research about the target community in relation to the application of a theoretical framework like the Health Belief Model for behaviour change [48, 49].

## Results

### Participants characteristics

Of the 41 women participating in the community focus group discussions, the average age was 29 years with a range between 20 and 38 years. The majority of women had attended primary school with or without some secondary education (41.5%) and 24.4% were illiterate or only experienced some primary schooling (see Additional file 3: Table S3).

All participants engaged well in the discussions. We present a summary of repeated ideas with selected representative quotes of the thematic areas explored.

### Thoughts on cervical cancer and its origin

Due to a lack of knowledge about cancer in general, as well as cervical cancer specifically, FGD participants - with and without medical background - could only make assumptions about possible signs and symptoms of cervical cancer. They are aware of many commonly known, however, unspecific gynaecological symptoms like pain, swelling, itching, burning and bleeding of the genital organ, as well as constant foul-smelling discharge. The clinic nurses were more precise in naming pathognomonic signs, risk factors and causes of cervical cancer, however only one of nine nurses mentioned the Human Papillomavirus (HPV) as causative factor and even among nurses and HDAL mainly cultural and traditional beliefs were reported when asked about the causes of cancer. One notably interesting aspect brought up consistently in all four FGDs was something called “**Mitat/Girefat**” in the local language. This means that “*sudden exposure of the body to sunlight*” – particularly while menstruating – “*sitting on hot surfaces*” and “*urinating on hot ground*” is thought to be the cause of many health problems. It is believed that urine evaporates once it hits the hot ground outside and the “*evaporated gas from the surface will carry disease causing agents to the inside (of) the body*”. ‘Girefat’ is a traditional concept for the pathogenesis of several diseases including sexually transmitted diseases [50, 51].

### Sequels of the development of symptoms

According to FGD participants, women affected by symptoms perceived as caused by cervical cancer would “*face stigma and discrimination*” by friends, neighbours and eventually even family members and marital partners leading to hopelessness and despair for the affected women. This was a particularly interesting aspect, as the fear of the social implications due to these signs and symptoms of a disease supposedly perceived as cervical cancer clearly outweighed the women’s fear of premature death connoted with cancer.

### The unfamiliarity of a screening concept

Despite the brutal prospects of falling ill as a member of the community, the large majority of women did neither know about the existence of cervical cancer prevention, nor would they necessarily seek it far in advance. The concept of screening – being examined for a disease that they do not yet suffer from but might in the future – is unfamiliar to them. Most people do not seek care unless they are already experiencing severe symptoms. Being able to fulfil their daily tasks and duties as farmers or housewives is much more important than worrying about their future health. A 26-year old community member said: “*In our community medical check-ups and early treatment seeking practices are no well-known issues. Even literate community members will not go to health facilities until the problem reaches a severe level*.”

Moreover, in the key informant interviews with a female midwife, female community health extension worker, as well as the male health official it became clear that screening is currently not offered in this health centre due to lack of facilities and equipment, even though local health professionals had previously been trained in providing the service. As there was no screening opportunity available, women of the community currently have low awareness nor previous screening experience. However, the midwife key informant stated: “*If the community (was) well aware about the disease they will have (an) interest to get the service*.”

### Barriers not to use preventive health services – fear

While further discussing preventive medical services, the topic of fear was brought up: Fear of needing expensive diagnostic work-up and treatment resulting in overwhelming financial charges. But furthermore, fear of being diagnosed with a disease with no treatment option available in their closer surroundings – regardless of finances – and consequently becoming an outcast of their community due to their diagnosis “*If there is no treatment after the examination, people will not want to be screened for cervical cancer*.”, said a 21-year old woman attending the health centre. This illustrates the perceived limited access to existing local, as well as national health services of remote and rural communities that might be derived from past experiences.

### Ease of use – the Evalyn® brush

After presentation of the self-collection procedure with the Evalyn® Brush the women throughout all four focus groups were positively impressed and confident to be able to perform the “task” of self-sampling after obtaining clear oral instructions. They were happy to see a simple device that will help them preserve their health for the future and bring health promotion from centralized and distant health centres closer into their community, making it more accessible for them.

However, one (26-year old) HDA leader was worried that women might blame the device in case they end up experiencing any gynaecological issues, as it “*touch(es) (…) sensitive area(s) of (the female) reproductive organ(s*)”. It was mentioned as an example that in the past Norplant® implanted for contraceptive purposes was made responsible for unrelated issues.

When asked about the place where to best implement the screening practice, perceptions among women of the community and women involved with health services were equally divided. Women who preferred home-based self-sampling listed advantages like alleviation of shame through privacy of their own house and increase in screening participation by overcoming some women’s inability to travel to the health centre, while on the other hand, women who preferred the health post or health centre for screening brought forward arguments like lack of hygiene at home with potential risk of contaminating the device, inability to bring the device back to the health centre, lacking medical proficiency to perform the “task” and lack of privacy at home.

Based on arguments of both sides, the focus groups agreed on the ability to perform self-sampling on the household level if women in the community were trained well in the performance of the procedure and mentored by local health professionals. However, the option of performing it at a local health post should be granted.

### Spousal permission – crucial barrier for HPV self-sampling

When probed about barriers for the implementation of community home-based HPV self-sampling, the overwhelming majority of women mentioned their spousal’s permission. As long as their marital partners were informed about the device and the exact procedure, the women would be granted unrestricted permission to undergo screening. Otherwise, they might associate the device with “forbidden sexual relation(s)”.

This patriarchal aspect of the Ethiopian societal system was confirmed by two male participants of the KIIs (see Additional file 2: Table S2). From their viewpoint, spousal permission is crucial and husbands should be well informed about the device in order to avoid enormous barriers for the women to harness this medical preventive service.

Therefore, it was strongly recommended by everyone throughout all FGDs and KIIs to create awareness, knowledge and education in the community about the disease cervical cancer, the self-sampling device itself and the associated opportunity of “task shifting” cervical cancer prevention directly into the community. This would simplify the implementation of a screening program comprising home-based HPV self-sampling tremendously.

## Discussion

HPV self-sampling is a promising tool to foster women’s health autonomy and to potentially widen cervical cancer screening coverage also to remote settings by shifting the sampling procedure to the community. Our study is the first to explore acceptability and barriers for home-based HPV self-sampling in a rural Ethiopian community with limited access to health services and naïve to screening practices.

Previous studies have shown that a multi-fold of women can be mobilized using self-sampling methods for cervical cancer screening in women all around the world [21–33, 52–55]. However, most studies are biased by recruiting mainly screening-experienced and interested women during a routine clinic visit or by inviting them to come to the health clinics to collect a sample themselves [33, 34]. This approach may exclude participation of hard-to-reach women who are already reluctant to attend medical services due to the logistical burden and expenditure of time that is entailed [20, 23, 29, 30]. In Europe, screening non-attendees were reached by mailing self-sampling kits to their home, however, these women have already had previous experiences with well-working health services and cervical cancer screening [21, 22, 24–27, 53]. Our study identified important themes to be considered when implementing home-based self-sampling in rural Ethiopia or similar settings.

### Awareness-building is key

A main concern is the lack of knowledge regarding cancer and cancer prevention. This was previously confirmed in studies by Birhanu et al., Getahun et al. and Mitiku et al. Despite it being proclaimed a health priority of the Ethiopian government [56] knowledge and awareness about cervical cancer is low in Ethiopian communities [48, 57, 58]. Several informants confirmed the existence of a traditional belief called “mitat” meaning ‘sudden exposure of the body to sunlight’ which is associated with the occurrence of cervical cancer.

In the absence of a clear concept of cancer most participants linked cancer manifestations with those of other major gynaecological diseases such as gynaecological fistula, prolapse and infections and emphasised the risk of social rejection, discrimination and stigma in severe cases. Education about women’s health problems thus seems to be an important component of cervical cancer awareness in order to make informed choices.

Education furthermore has the potential to mitigate feelings of “embarrassment”, “shame” and “fear”, important barriers for women to refrain from a preventive gynaecological examination as shown in Western communities [59–62], as well as in communities in Zambia and Uganda [63, 64]. White at al. showed that these psychosocial barriers were the most modifiable of all barriers as they are “dynamic” rather than “static” emotions because they decreased over time once women were educated about cervical cancer – especially about the fact of early detection, slow progression and treatability [64]. In a study by Crofts et al. teaching 540 women in Cameroon about the Human Papillomavirus and cervical cancer achieved higher acceptance of HPV self-sampling and a decrease in embarrassment, anxiety and discomfort [35]. Teng et al. noted that the overcoming of these psychosocial barriers might have even greater benefit for the uptake of screening than the immediate abolition of socio-economic, financial or logistical barriers [63].

### Increasing women’s confidence

Initially participants were concerned regarding their ability to perform the test correctly [43, 62, 65–67]. However, explaining the procedure using a ‘demonstration brush’ and a description video assured the participants about their capability to perform self-sampling.

Hanley et al. provided 200 eligible Japanese women of which merely 22.7% had previous experience with tampon use with just written and pictorial instructions on a self-sampling kit [43]. Nevertheless, 96.9% had no trouble comprehending the instructions. Some of the participating women were unconfident that the test was administered correctly in comparison to a physician-collected sample, while in reality no invalid samples were submitted. Similar findings were reported in a study by Vanderpool et al. in a population of rural Appalachian women [68] and by Sewali et al. in a population of Somali immigrants in the US [69]. It hints at findings of the previously mentioned study by Crofts et al. in Cameroon where through prior education 90% of women were confident to have performed self-sampling correctly and 96.7% found it easy to perform [35]. Provision of instructions appropriate to each study population (literate or illiterate) and information on the accuracy on HPV self-sampling will increase women’s confidence in their ability to perform the test correctly [70]. This also confirms findings of other studies in several female African populations showing that women are indeed willing to collect their own cervical samples and that self-sampling methods are socially acceptable and possibly even more feasible than other screening methods like Pap cytology or visual inspection with acetic acid (VIA) [71–73].

### Including the family

Crucial family members – especially the women’s husbands – need to be informed and involved in the process in order to avoid mistrust and misunderstanding. Our findings show that – if well informed – they will endorse their spouse to test, preserve and improve their health status. They were also accepting the self-sampling device as long as confidentiality and privacy of the woman are ensured to prevent stigma and discrimination. Likewise, Mutyaba et al. determined they are “potential willing partners” in improving women’s and maternal health [74]. Sewali et al., moreover assessed that women having friends or family members with whom to discuss cancer screening were three times more likely to participate in any screening test than those who do not [69].

### Importance of care continuum

The concept of secondary prevention, especially the fact that supposedly “healthy” persons have to undergo a medical test, was unknown to most community participants. In order to promote “task shifting” of cervical cancer screening in the communities, explanation of the screening aim to early detect precancerous lesions and the slow progression and high potential of cure of these lesions are an important part of the information package. Women further stated the importance to be assured about treatment availability in their surroundings to raise credibility, reliability and trust in the local health services. We found consensus in all four FGDs regarding the potential role of the existing health network (CHEW and HDAL) in promoting, disseminating and performing home-based self-sampling as part of the WHO’s “task shifting project” either in the home or when preferred in the health post. The study is an important activity to prepare a community-wide HPV-based screening and treatment campaign in the region as it is a major uncertainty whether self-sampling on the household level would be acceptable.

### Limitations

As the study findings are based only on small samples, they may not be generalizable within Ethiopia, let alone the whole sub-Saharan African region due to socio-cultural differences between regions and countries. Furthermore, the research team was limited in its ability to explore the perspective of all ethnic groups within the target community.

Some questions posed to the groups were necessarily speculative in nature, since the proposed program does not yet exist; it is therefore difficult to predict reactions to a program once it is implemented.

## Conclusion

Home-based self-sampling for cervical cancer screening is a socially acceptable and feasible “task shifting” method that could increase cervical cancer screening access and coverage in the Ethiopian study community. Education, community mobilization, awareness creation and family inclusion are identified as key activities to promote, implement and facilitate self-sampling in rural and low-resource African communities. Already existing social networks, for instance religious communities, and peer-to-peer education could be useful in passing down information on health-related topics, including HPV self-sampling and increasing women’s confidence in being able to perform this “task”. Husbands and other crucial family members play key roles in women’s health behaviour and therefore must be involved early in order to avoid mistrust.

## Additional files

Additional file 1: **Table S1.** Distribution of participants in community Focus Group Discussions (FGD). (DOCX 47 kb)

Additional file 2: **Table S2.** Key Informant Interview (KII) Participants. (DOCX 43 kb)

Additional file 3: **Table S3.** Characteristics of Focus Group Discussion participants (DOCX 46 kb)

Additional file 4: English language copy of the Interview and FGD Guides used as supplementary files. (PDF 396 kb)

## Data Availability

The datasets generated and analysed during the current study are not publicly available due to possible inclusion of identifying confidential personal data but are available from the corresponding author on reasonable request.

## Abbreviations

(hr)HPV: High-risk-type Human papillomavirus
CHEW: Community health extension worker
FGD: Focus group discussion
HDAL: Health development army leader
HIV: Human Immunodeficiency Virus
HPV: Human papillomavirus
IRB: Institutional Review Board
KII: Key informant interview
LMIC: Low- and middle-income countries
OGAC: Office of the United States Global AIDS Coordinator
VIA: Visual inspection with acetic acid;
WHO: World Health Organization
