# Supplementary material for: Genital self-sampling for HPV-based cervical cancer screening: a qualitative study of preferences and barriers in rural Ethiopia
Source: BMC Public Health. 2019 Jul 31;19:1026. doi: 10.1186/s12889-019-7354-4 (PMC6669971; doi:10.1186/s12889-019-7354-4)
Supplement: Supplementary file 1 — Table S1. Distribution of participants in community Focus Group Discussions (FGD). (DOCX 47 kb) [file 12889_2019_7354_MOESM1_ESM.docx]

**Additional File 1: Table S1**

**Table S1 – Distribution of participants in community Focus Group Discussions (FGD)**

| **Focus Group** | **FGD Type** | **Females (total)** | **Age range** |
| --- | --- | --- | --- |
| FGD 1 | Health Centre Attendees | 8 | 21 – 38 years |
| FGD 2 | Health Development Army Leaders * | 10 | 24 – 34 years |
| FGD 3 | Nurses at Health Centre * | 9 | 25 – 38 years |
| FGD 4 | Members of the Community | 14 | 23 – 28 years |
| FGD 1-4 | All of the above | 41 | 21 – 38 years |

* indicating participant with medical background
