# Supplementary material for: Genital self-sampling for HPV-based cervical cancer screening: a qualitative study of preferences and barriers in rural Ethiopia
Source: BMC Public Health. 2019 Jul 31;19:1026. doi: 10.1186/s12889-019-7354-4 (PMC6669971; doi:10.1186/s12889-019-7354-4)
Supplement: Supplementary file 2 — Table S2. Key Informant Interview (KII) Participants. (DOCX 43 kb) [file 12889_2019_7354_MOESM2_ESM.docx]

**Additional File 2: Table S2**

**Table S2 – Key Informant Interview (KII) Participants (n=4)**

| **Key Informant** | **Individual Type** | **Gender** | **Age** |
| --- | --- | --- | --- |
| Key Informant 1 | Husband of patient attending Dabat Health Center | Male | 45-50 |
| Key Informant 2 | Midwife at Dabat Health Center * | Female | 30-35 |
| Key Informant 3 | Community Health Extension Worker * | Female | 30-35 |
| Key Informant 4 | Dabat Health Center Official * | Male | 30-35 |

* indicating participant with medical background
