# Supplementary material for: Genital self-sampling for HPV-based cervical cancer screening: a qualitative study of preferences and barriers in rural Ethiopia
Source: BMC Public Health. 2019 Jul 31;19:1026. doi: 10.1186/s12889-019-7354-4 (PMC6669971; doi:10.1186/s12889-019-7354-4)
Supplement: Supplementary file 3 — Table S3. Characteristics of Focus Group Discussion participants (DOCX 46 kb) [file 12889_2019_7354_MOESM3_ESM.docx]

**Additional File 3: Table S3**

**Table S3 – Characteristics of Focus Group Discussion participants**

| **Characteristics** | **Number of Participants** |
| --- | --- |
| **Age groups** |  |
| 20-25 | 11 |
| 26-30 | 15 |
| 31-35 | 10 |
| 36-40 | 5 |
| **Total** | 41 |
| **Education** |  |
| No schooling or some primary schooling | 10 |
| Primary with or without some secondary schooling | 17 |
| Secondary | 3 |
| Postsecondary | 11 |
| Total | 41 |
