# Supplementary material for: Genital self-sampling for HPV-based cervical cancer screening: a qualitative study of preferences and barriers in rural Ethiopia
Source: BMC Public Health. 2019 Jul 31;19:1026. doi: 10.1186/s12889-019-7354-4 (PMC6669971; doi:10.1186/s12889-019-7354-4)
Supplement: Supplementary file 4 — English language copy of the Interview and FGD Guides used as supplementary files. (PDF 396 kb) [file 12889_2019_7354_MOESM4_ESM.pdf]

FGD and KII Topic Guide to understand barriers of Self-Sampling

Group participants:

- **Women attending Dabat Health Clinic** (for female, maternal and child health, chronic diseases, acute infections, etc.)
- **Health Development Army Leaders (HDA Leaders)**
- **Members of the Dabat Community (Women)**
- **Nurses of Dabat Health Clinic**

Group size: 8-10

Number of groups: 4

In-depth Interviews: 4 Key Informants

- 1 Health Center Official
- 1 Health Extension Worker
- 1 Midwife
- 1 Husband

Items included into the focus group discussion guide:

- Cervical cancer perception
- Cervical cancer screening awareness
- Embarrassment: sampling, handling of sample, doing sampling correctly
- Fear: finding cancer, causing cancer, perception of others
- Spousal permission
- Body knowledge
- Assisted sampling
- Male vs. female healthcare worker
- Does parity reduce barrier?
- Home sampling vs. clinic sampling
- Barrier of transportation to clinics / doctors

Questions on Cervical cancer Knowledge and Awareness:

1. What do you know about cervical cancer?

2. Have you, a family member or anyone in your community been affected by signs & symptoms of cervical cancer (e.g bleeding after sexual intercourse, pain during sexual intercourse, foul smelling discharge)

3. How is the perception of someone affected by cervical cancer?

4. Do you know what causes cervical cancer?

    a. Virus

b. Bacteria
c. Traditional beliefs
d. Other

Questions on cervical cancer screening:

5. Is there a cervical cancer screening opportunity in this district?

    a. What is the cervical cancer screening opportunity in this district?
    b. What is your experience with cervical cancer screening?

6. What are facilitators for you to go to cervical cancer screening?

7. What are barriers for you NOT to attend to cervical cancer screening?

    a. Fear
    b. Embarrassment
    c. No permission by husband / spouse
    d. No clinic / nurse available
    e. Socioeconomic Factors
    f. Cultural and Religious Factors

8. Who do you prefer to do the examination (Health Professional or yourself)?

    a. If you prefer Health Professionals – Where?
        i. Health Center
        ii. Health Post
    b. If you prefer Yourself at home
        i. Is there a private place available in your home?

Questions on Self Sampling Device – the Evalyn Brush:

Explanation of the Evalyn Brush by Dr. S. (M.D. and Gynecologist, UoG)

9. What do you feel about this procedure of this device?
    a. Could you easily use it at home? (Self Sampling)
        i. with WRITTEN instructions in Amharic
        ii. with VERBAL instructions in Amharic (for illiterate)
        iii. Would you prefer someone / a FEMALE to help you with the self sampling, e.g. explain to you how it works
    b. Could it be socially accepted by (all or) most women in the community?
    c. What might be barriers, problems and concerns about this device for you and other women in your community?
    d. What do women consider as barriers NOT to use the device (permission of husband or spouse?)

10. How do you think our Self-Sampling-Project could be feasible in your community? (at Home-Level or at Health-Post- or Health-Center-Level)

FGD-1 Transcription

Participants: **Women Attending Dabat Health Center**

Moderator: Mr. S. H.

Starting Time: 8:50 AM

Ending Time: 10:05 AM

FGD Setting and process: Eight women came together in the health center. They were sat in a circle in order every one can easily discuss and see each other. After the overall information was given, the FGD participants were asked their willingness to participate in the discussion as well as for tape recording. They all agree on participation and tape recording. During discussion FGD guide was used, note taking and tape recording were done.

Demographic characteristics of participants in the discussion

S. No	Code of the participant	Sex	Age range	Level of education	Position in kebele/district
1	M-01	F	30-35	Illiterate	No
2	M-02	F	30-35	Grade-6	1 to 5 leader
3	M-03	F	20-25	Grade-10	No
4	M-04	F	20-25	Grade-10	No
5	M-05	F	20-25	Illiterate	No
6	M-06	F	35-40	Illiterate	No
7	M-07	F	30-35	Grade-4	1 to 5 leader
8	M-08	F	20-25	Level-3 Diploma	no

Theme 1: Knowledge and perception on cervical cancer

Question 1: Knowledge on cervical cancer

A 21 years old women said that "first of all cervical cancer induce strong pain sensation on genital organ and after this foul smelling discharge may come out of the body. This will continue

to cause severe pain in the vagina. During this we can say that women are exposed to cervical cancer problem."

A 24 years old women said that, "when this problem (CC) occur it has symptoms like swelling and itching of genital organ. Which results in wound on and around genital organ’’

A 30 years old women said that, "CC is a health problem the occurs on females and have symptoms like bleeding for long period of time, discharge from the body, burning sensation and pain on genital organs’’

A 35 years old women said that, "I came from rural area we don’t know CC specifically. However, if we face problem we will go to health center for examination and treatment. I don’t know what CC is as a health problem’’

Question 2: Anyone who experience the problem in your community

A 24 years old women said that "I have seen one women with such sign and symptom. First of all early marriage was the cause for her problem. Then after she says that she feels pain during inter course itching near genital organ, and foul discharge starts to came out of the body at the end she got gander university hospital and know she have got relate."

A 24 years old women said that ”in our community I this difficult to discuss such sensitive issues. Women will not expose such problem to others. This condition was dominated among those women who live in rural areas. So we only tell rear condition that we hear’’

A 30 years old women said that I have seen one women in my neighbourhood. She always claim continuous vaginal bleeding. Burning sensation and repeated spontaneous abortion I think this are signs o cervical cancer.

Question 3: How is the perception of the community on someone with CC?

A 38 years old women said that ‘’ the first the will isolate themselves from their family as well as society; the four smelling discharge will not allow them to equally interact with other community members. The community may also isolate them by fearing the foul smell. The other thing they may be embarrassed to be with other people while having this smell. This result self-isolation first and follow by others is oblation.

A 35 years old woman said that ‘’ this problem may result separation of a married couples. Especially when she close her problem & didn’t get treatment men’s will not be voluntary to continue with them’’

A 21 year old woman said that “If she is no healthy she can’t life with her husband. The foul smell from discharge also make him to lost for other women, so this problem may end with separation of married couples’’

A24 years old women said that ‘’this health problem will change the behavior of the women. They will not have a good behavior. They start to health and collide with other people s including family members. They will start maundering in their house. So they will have distinct behavior them.

A 30 years old woman said that “the isolation they encounter will make them to be hopeless in their future life. They start to think about their death and break down of their family .they will not have peace in their mind and always feel disturbed.

A 24 year old woman said that “as my friend say above women with such problem will not have peace and love with their family members as well as with their neighbors most of them will not think about getting relief from treatment of such problem.

A 30 years old woman “they will become hopeless. They start to ask they god to end their life. They will not speak this problem to anyone”

A 35 years old woman said that “women with such problem will prefer to be a one for long period o time. They may go to health facilities for treatment if they will not get relief they sit alone until their date of death “

Question 4: causes of cervical cancer

A 20 years old women said that “the major cause of cervical cancer is early marriage. when women enter in marriage at early age during sexual intercourse lesion may occur on genital organ. If these continue for a period o time in will be changed to cervical concern. The other cause is the wound that occur after delivery process. If this wound will no teal for longer time it will result in cervical concern”

A 24 years old woman said that “when women sit in the sunny time while they are on menstruation this problem may occur which is called “mitat” in local conjugate additional to the early marriage also will cause this problem. Such women will have developed body for sequel inter course. So during inter course their genital organ may lacerate which results in cervical cancer”

A 30 years old woman said that “according to me the other cause of cervical cancer is delivering birth at home by assistance of traditional birth attendance (TBA). This TBA will not have adequate training to assist birth. While they wait for normal delivery prolonged and obstructed lob our may occur. This will cause Cc in the future late of the women.

A 21 years old women said that “sudden exposure of previously cohered body to the sunlight locally called “mital/geretita “cam cause Cc. this may occur when will sit on hot grounds and urinating on hot ground. The propagating no their will cause such problem. On the other hand prolonged sexual intercourse will all result this problem.

Theme -2 Question on CC screening

Question 5: CC screening opportunity in the district

A 30 years old women said that “A cervical cancer screening service will not be given in our local area .I them it will e give in gander specialized hospital. Because of this we haven’t got the screening service until now”

A 24 year old woman said that “we don’t know specific cervical cancer screening procedure. However, any women will came to health facility for examinant on and treatment. They may also retired to gander & debark hospitals. Still we do not know the exact name of their health problem as well as the screening”

A 35 years old women said that “I haven’t hear service cancer screening service for women in our locality .so I think the service will not be given in our date health center”

Probe: What is your experience with CC screening ?

A 24 years old women” still we will not have screened for cervical concern. We will come to health center when we have certain health problem only .so I we say even we do not know the existence of the service”

Question 6: Not Eligible

Question 7: what are the barriers for you not to be screened?

A 30 years old women “the major barrier for not we screened for cervical cancer is fear to express the problem to other .the other factor is being embarrassed to disclose the problem to each other”

A 21 years old woman said that “we do not know the problem cervical cancer as well as the screening service available to distract the problem. So, this lack of awareness is the barrier not to be screened for CC. Lack of knowledge make women to be embarrassed to communicate about the problem."

A24 years old woman said that “as the previous speared said, each of knowledge may be the cause for not to be screened. But this is no to mean all community member are free from CC. they may have the problem”

A 30 years old women said that “some women will fear examination charge to get the screening service. While the others will feel embarrassed to expose their problem to others. I think if all community members are a worker of this service. They will come and use it “

Probe: What should be done to initiate the utilization of screening service?

A 38 year s old women said that “what your are saying for use is new of we do not how such awareness first. If all women in our area hear about this issue the will came to health center. so creating awareness should precede he service after that not of the community members deny the service .

A 24 years old women said hat “most women will not come to health facilities unless they are unable to do their daily late activities, as a result most of them will not consider them salve as at risk of encountering the problem. This will hinder utilization of the screening service; this

condition can be change by having strong health communication at health center as well as at community level.

A 21 years old woman said that “the other thing is that, most of us fear availability of drug/ treatment / after screening process. If there is no treatment after examination people will not want to be screened for CC.

A 24 years old woman said that “sometimes woman may not get permission from their husband to go to health center for screening. This is another barrier for utilization of the service,

Probe: facilitating factors to us the screening service.

A 30 year old woman said that “to initiate the screening service first of all health information /education should be given to all health development army teams and again the information should be transfer at all community meeting at different levels’

Questions 8: who do you prefer to do the examination? Where?

A 21 years old women said that” If there is an instrument that we all can use to test the problem, it will best if we perform it at home. if there is no such instrument if can be done at health institution .

A 24 years old woman said that “even if we have such instruments, we have lower knowledge that health professional. So from my own point of view it will be better if we take the screening service form health professionals”

Probe: For those who prefer self screening; is there a private place in your home?

A 21 years old women said that “if the instrument if given to us and if it is simple to use we can do it at home. It is not difficult to arrange private time of place to undergo self screening”

A 21 years old women said that” first of all I should get permission from my husband. If I tell all the situations he will accept my concern, after this it is my responsibility to arrange time and place in my home to take the sample so ,I can arrange this in my home”

A 30 years old women said that “I prefer to be screened at health facilities because have everything is clean, there are trained health professional and there examination coach which

facilitate the screening procedure. We cannot have all those things at home .so I think it is better if it will be done at health center”

A 38 years old woman said that “I do not know anything about such medical procedures and self sampling practice so, it will be better if it will be done of heath center by knowledgeable health professionals’’

35 year old women said that “I think women in the town or educated women may able to undergo self sampling procedure at home however, those women like my who comes from rural areas it is nearly impossible to do self sampling at home, like other health service such as immunization family planning it will be better if it will be done at health center, each of knowledge and educational status of the woman will influence such activity.

24 years old women solid that “if I can do it at home, I can take the procedure in my home, this is only if the procedure is simple for me”

A 30 year old woman said that “to do self sampling we need strong training on how to do self sampling but after we take training and know the procedure it will be easy for us to do so,”

Theme-3 Question on self sampling device

Question 9: what do you feel about this procedure of the device?

A 24 years old woman said that “the device as well as the procedure looks simple to undergo self sampling. I will not require any reading instruction. So, there is no problem with the use of this device for self sampling. Specially it is will be simple for latent women’s”

A 24 years old woman said that “the device looks simple to use at individual level .If it is given for us, it is simple to undergo self sampling.

A 38 years old woman said that “If it is like this it is very easy to undergo self sampling, every women can perform this self sampling procedure after clear oral instructions’

A 21 years old women said that “as we see from the demonstration It looks simple procedure. So every woman can do so but, creating awareness should be dome first.

A 30 years old woman said that “this is very simple procedure for most of us. As said above, prior to implementing the self screening program you need to work on health education /crating awareness, so I will best.

Probe: What might be barrier, problem and concern about this device?

A 24 years old woman said that “there may be husband that may collide with his wife if she didn’t tell him about the procedure and the device”

Probe: would you need any to help you in self sampling?

A 38 years old woman said that “If what we see is the exact procedure, it doesn’t require any one to assess as in self sampling process.

Probe: Social acceptability

A 24 years old woman said that “none of the community members will no denial this procedure as well as this device”

A 24 years old women said that “In my opinion this device will not face any barrier from the community. As you have demonstrated for us it is better to do so for all community members. After this they will accept and use it,”

A 21 years old women said that “ I think this will no requiem educational level and related competences been illustrated women’s can use it by looking it only, if the brush will be distributed after education I given for them every women can do self-sampling procedures,”

A 24 Years old women said that “In m opinion this is to know our heath status/condition. So it will not face any problem in the implementation,

35 years old women “all women will accept it for the sake of their health condition. So I haven't seen any potential barriers or concerns that influence this procedure.

A 38 years old women said that “ still it may be difficult for rural people who are illiterate to lake and use the device. For example, if I take this to my home all will ask me by saying what o you bring to us “ additionally it is new to community members .so it require creating awareness before you start the process.

Probe: What do women consider as barrier not to us the device?

A 30 years old women said that “in my opinion this procedure should be done after we communicate with our husband & family members about the device. I can take the same by myself, but I soul asked permission to do so from my husband.

A 24 years old woman said that “If my husband look the device before informs him about it, he may collide with me by thinking about other things. He can no positively evaluate the device as she only use for screening purpose, however, if I tell him all thing he will permit for me and I will also became happy ,if I do so.

A21 years old women said that `It will be better to inform our husband about the benefit of the device and he exact procedure before we start safe sampling .Then he will allow us to do so and self sampling become easy for us.`

A35 years old women said that ` Other than husband permission .There will be nothing that can be barrier for this self sampling procedure.

Probe: If it is at health center who do you prefer to assign self sampling?

A24 year's old woman said that `If it is at health center it will be best than self sampling At Health center all are health professionals. So we have no preference items of sex.`

A30 years old women said that "we are from rural areas even we feel embarrassed when male health profession comes to assist delivery process .They fear to expose themselves to male health professionals. So if possible we prefer female health professional to assist self-sampling procedures.`

A24 Years old women said that ` we may feel embarrassed to expose our Selves in front of male \health professional So I prefer if it will be done by female health professionals.

A20 Years old women said that `I will not feel anything in front of health professionals. Both females and males are equal for me ,But women in rural areas may prefer female health professionals than male .But there is no problem one of them can assist as in self sampling.`

Question-10- How do you think self-sampling project could be feasible in the community?

A24 years old women said that ` The comminuting will give attention if the producer is done at health center .But if it is at home level the community may look it as joking .Sometimes people may forget things as home ,so it will be best if it is done at health center.

A21 Years old women said that ` I also prefer if it is at health center. Because WR all may not equally use the device appropriately at home some may not keep it in clean place. Some may forget it. So to avoid all this issues it should be at health center.

A35 Years old women said that ` I Think if possible it should be at health center. Because many people will have no confidence on self sampling some may respect the idea .But if it is at health center they give respect to the procedure and it will be effective .`

A24 years old women said that ` The community may not belie  self- sampling procedure .Even health extension worker will not have equal respect and credibility in front of community member .If it is at health post they may ignore the producer .But ,If it is at Health center everything become effective`

A30 years old women said that` Most community member are still illiterate It is difficult to communicate effectively and accepted by them with short period of time. We need to use already accepted institutions and professionals at health center. Health extension worker will also encounter certain difficulty on this process than health professional. So it will be better to give the service at health center.

A24 Years old woman said that ` If it is at health center we can mobilize women to use the service by our health development any team .Everyone will hear the massage and health professionals will help in sample collection.

FGD-2 Transcription

Participants: **Women Members of Dabat Community**

Moderator: Mr. S. H.

Starting Time: 1:40 PM

Ending Time: 2:55 PM

FGD Setting and process: Twelve women came together in the health center. They were sat in a circle in order every one can easily discuss and see each other. After the overall information was given, the FGD participants were asked their willingness to participate in the discussion as well as for tape recording. They all agree on participation and tape recording. During discussion FGD guide was used, note taking and tape recording were done.

Demographic characteristics of participants in the discussion

S. No	Code of the participant	Sex	Age range	Level of education	Position in kebele/district
**1.**	W-01	F	20-25	Illiterate	No
**2.**	W-02	F	25-30	Grade-9	1 to 5 leader
**3.**	W-03	F	25-30	Illiterate	No
**4.**	W-04	F	25-30	Grade-4	No
**5.**	W-05	F	25-30	Grade-8	1 to 30 leader
**6.**	W-06	F	25-30	Grade-8	No
**7.**	W-07	F	25-30	Illiterate	1 to 5 leader
**8.**	W-08	F	25-30	Grade-5	No
**9.**	W-09	F	25-30	Grade-10	1 to 5 leader
**10.**	W-10	F	20-25	Grade-10	No
**11.**	W-11	F	25-30	Grade-10	No
**12.**	W-12	F	20-25	10+2	No

Theme 1: Knowledge and perception on cervical cancer

Question 1: Knowledge on cervical cancer

A 25 years old woman stated that | I have seen one women with such problem in our community .now she was treated at university of gander hospital and she became health .this cervical cancer will have similar facture with fistula”,

A 30 years old woman said that “I have seen one woman with this problem. She complained that fault smell discharge come out her vagina. But she treated at gander hospital.”

A 26 years old women said that “we do not know such health problem before. As a result we haven’t asked such symptoms among women while discussions o health issues. But after this if we ask them we may get evident with such problems”

27 years old women said that “I have seen one woman in my neighbor who complains whit discharge came two of her body after urination and server lower abdominal point. This discharge will have foul smell to me .I them these signs are indicator soft cervical cancer ‘

A 34 years old woman said that “we have no idea about cervical cancer it is new disease for us “

Question 2: Anyone who experience the problem in your community

A 28 years old woman said that “this sign and symptoms are known to us. So we haven’t heard any one with such problem in our community. But if we tell such sign of stomps to them they may disclose their health problems.

A 27 years old woman said that “previously foul small discharge is a sign for Laval transmitted infection. So, nest of the sign and symptoms of cervical cancer are similar with other STI when people look the signs they will go to health center and take drug for their problem. So we consider such problem as sit rather that cervical cancer,”

A 24 years old woman sail that, “I have one neighbor who complain for foul smell discharge from her body. But she will not have sighs like bleeding after inter course and point during sex. Then she go to health center for treatment but she didn’t get any treatment there. She was only advised to keep her personal hygiene properly.”

A 26 years old woman said that “for example, I feel pain during sexual inter course, with my husband, I came to health centre but hey simply gave me painkiller for headache. After taking that drug I can get relief from my problem. Still I feel point during sexual intercourse,”

A 25 years old woman said that “in our community there is no awareness about cervical cancer. Beforehand, there is no such knowledge on this problem. So no one will discuss this as an issue at community 1to5 gathering,

Question 3:- How is the perception of the community on someone with CC?

A 30 years old women said that “If she have four smell discharge people my isolate her. Her husband also will not be voluntary to live with such women. He will go other woman; this will result in separation of the married couples. The will unable to stay together, so it will result difficult social problem,”

A 28 years old women said that “for example if I have such problem my neighbors will no want to be with me .even my husband may hate me .he don’t want to have intercourse with me. This make him to find other healthy women, the community also ignore and isolate me to be alone.

A 25 years old women said that “women with such problem will lose their self . Confidence and feel loneliness, they will prefer to be alone”

A 26 years old woman said that “I have seen one women with cervical concern problem. Now she will unable to control her urine. She will stay at home throughout the day. She also gone to Gander University specialized hospital, but they will say you came at late stage. New day she will live at her home alone. The foul smell willing tallow any one to enter her home. Hew every. If this woman discloses her problem immediacy at early stage she may get appropriate treatment.

A 24 years old women said that “our community have latch of knowledge on this regard women with such problem will face stigma and incrimination. They will not have a person to accompany them and like alone for long period o time.

Question 4: causes of cervical cancer

29 years old women said that in my opinion poor personal hygiene is one of the causes of cervical cancer. If women will have good personal hygiene the disease causing agent will entry in to their body during sexual inter course. This will result cervical cancer on her,”

A 30 years old women said that “sitting on hot material or surfaces will expose us to cervical cancer who on is called mitt/gin fat “in local language.

A 37 years old women said that “according to me prolonged lab our will cause cortical concern. For example, If I take my own problem, before three year during lab our I was on the way to health center by compliance car. In the mid-time the now born came, but the driver didn’t stop the car for me starting that time I will go to health center for the case of point during inter course but ,they didn’t treat me accordingly. So, I face this problem as a result of prolonged lab our history in the past,”

A 25 years old women said that “sitting on ho surfaces, lack of personal hygiene and not using condom during inter course. This will cause cervical cancer. Especially sitting on hot surface of urinating on hot ground will result is what socially canoed “mitt/parental” which will cause cervical cancer.

A 25 years old woman said that “In rural community the cause of many health problems is this sudden exposure of the body to sun light or sitting on hot surface; which is called “Mitat/Girefat” in local language “?

A 27 years old woman said that most of the time this problem will occur among female commercial sex worker; because we will have intercourse with many different customers. The other major cause of cervical cancer is lack of personal hygiene among women additional women in our community urinate at any place they get. During this time the evaporated gas from the surface will carry disease causing agents to the inside body. This will result cervical cancer,”

Theme -2 Question on CC screening

Question 5: CC screening opportunity in the district

A 26 years old woman said that “most of the times we hear about serve transmitted diseases screening and treatment. We have no idea about the existence cervical cancer screening service

in our district. Additionally in the previous few years we have heard about fistula treatment. However, the issue of cervical concern is new to us,”

A 20 years old woman said that “wham women face such signs and symptoms. Most of the time it is considered as STI or fistula, but still we haven’t hear about cervical concern screening service similarly we all will not have the experience of CC screening.”

Probe: What is your experience with CC screening”

A 35 years old woman said that “until now I have no experience on CC screening, I haven’t hear from any one in my neighbor about the service as sells its utilization.

A 30 years old woman said that “most of us or morally all of us are mew even to this type of health problem, so, we don’t know the availability of the screening service .and we will not yet screened for cc as a health problem.

Question 6: Not eligible

Question 7: what are the barriers for you not to be screened?

A 26 years old woman said that “I think if we can do our daily life activity properly we will not got health facilities even if we face certain discomforts. We were not go to screening because we will expect that the problem will occur on us. Out if we unhealthy we will go to health center for examination,

A 24 years old woman said that “women will fear to expose such problem to theirs they may stay for long period of time with the problem, this is because of fear and difficulty to expose such issues for others,”

A 30 years old woman said that “most women will have a doubt about the existence of appropriate treatment if the problem occludes on them. If they think they are unable to get the treatment they may prefer not to be screened from the initial. They will also fear the disclosures of their result to their neighbors. if once their status is known the information will reach the community member if she will unable to get treatment if became a source of isolation.

A 26 years old woman said that “the other issue is waiting permission from husband. it is mandatory to get permission from husband to undergo the screening process. For simple case even we will ask permission from them for example today to be part of this discussion a came after he permit for me .so this procedure is more difficult them simply attending meeting his permission is very important. Same times he may prohibit no to undergo such tests. this was one of the barriers for no to be screened.

A 24 years old woman said that, “most women will not come for screening service. Because, they will not get appropriate treatment for their health problem. When they go to health center the always give tablets and injections which will not change their health status. I am evidence for this I came to health center for more than 2 times .but I can’t get relied from the treatment center for more e than 2 times. But I can’t get relief from the treatment I recovered.

A 26 years old woman said that, “most of the time people will no easily accept and use such screening service. They fear health problem as well as shortage of many to undergo screening. They fear knowing the exact health status and existence of a treatment for it,”

A 26 years old woman said that “ In our community medical checkup and early treatment seeking practices are no well know issues, even literate community members will at go to health facilities until the problem reach severe level. This is another barrier not order go screening service.

Questions 8: who do you prefer to do the examination? Where?

A 24 years old woman sailed that “I will prefer to be examined by health professional at health center. If it will be done at health center your will also have follow up if you have a problem,”

A 26 years old woman said that “women’s will be embarrassed to be exposed inform of others. For this reason it is better to undergo the examination at individual level at home “

A 30 years old woman said that “among community member, embarrassment is common on sexual issues. So, if possible self–examination will avoid this condition .but; this will require giving arraigning for community members prior to starting the program. “

A 35 years old woman said that " I have different idea with my friends I prefer to be examined at health center by health professionals. if it is at individual level we any no appropriately perform the procedure. we may fear being exposed to other family members. so if possible I prefer health center. for nearness we can use health poses and health extension workers,"

A 29 years old woman said that "I prefer to be examined at home. I can arrange private place and time to take sample. From my body,"

A 25 years old woman said that "in my opinion I prefer to take the service at health center. I few try to apply it at home it will be difficult to appropriately using if. We may feel embarrassed and fear other family member if they look during self sampling.

Probe: Is there private place for you're to undergo self sampling?

A 26 years old woman said that "when we see things ideally it may seem simple to apply. Most community member's life in single class house, it will be difficult to arrange private place for self sampling. so it will be better if it will be done at health center.

Theme-3 Question on self sampling device

Question 9: what do you feel about this procedure of the device?

A 27 years old woman said that 'the device as well as this procedure looks simple and any one can perform similarly after observing demonstration. As I have seen it a cam also takes my sample easily at any time,"

A 23 years old woman said that, "I am happy to see such a simple device that will help us for cervical cancer screening. it will be done with our touching the upper surface of the device. This clearly avoid any contamination that may occur during screening,"

A 24 years old women said that, "as we all looked, it is simple to take sample from the body, and many women also can do this sampling procedure at home, it will not require formal education."

Probe: Could you easily use it at home?

A 25 years old women said that, " I think I will be better if the screening will be done t health post, If it will done at individual level people will not give attention to it and they may forget of

undergo the procedure, same people may fear side effects like if may cause certain ill turns on them.

A 27 years old woman said that," I prefer if the procedure will be done at health center after aerating awareness about the device, we can also participate on creating awareness activity at health development group level.

A 26 years old woman said that," In my opinion it will be better if it will be done at home by individuals. It is so easy and any one can perform I throw ever, after taking the sample we may forget to bring I appropriate time to health centrist may also disappear or children at home make take to play within and other problems may occur. To avoid such issues it will be better to arrange the screening at health posts,"

A 25 years old woman said that "as we say before the procedure is very simple and cam do it at individual level. If possible it will be better if it come 5 with clear written instruction for literate individuals. After that you can take the sample at home while taking a path or when you are alone at home,

Probe: Would you prefer someone to help you with self sampling?

A 26 years old woman said that," according to our community we will discuss everything with our husband before with engaged in the situation. Similarly my husband can also assist me in this self sampling process,"

A 25 years old women said that," according to me, I don't need any support to take sample from my body. Including my husband. I can do it by myself."

A 30 years old women said that ," there are a lot of women who will no have husband and child. In May opinion they can take the sample themselves without any assistance"

A 35 years old women said that "I think it will be better if I take the sample for mu self. I may not wait for my husband permission. In my bed room 1 take the sample wham I am a one at home.

A 24 years old woman said that,” the procedure will take maximum 5 minutes which very short period of time. we came o this at home after closing our door and the we can bring to health center ,no one will feel difficulty in doing so.

Probe: Could this device and procedure be socially acceptable in the community?

A 29 years old woman said that “I think one device is used for one woman only. His will avoid the potential mistrust that may associate with re use of the device .so they will accept and use it. This is for their own health.

A 24 years old women said that,” first of all we need to work on creating awareness about the device and the procedure. If the understand this issues appropriately the will definitely accept it,’

A 26 years old woman said that “we have 1 to 5 as well as 1to 30 group arrangements in our community. We have regular meeting time to discuss about or health status. During this time we can create awareness about the device for women in our groups. However, if we directly ask them to undergo sampling they may dining as,”

A 38 years old woman said that “ now we got a lot information about the device and it benefit .as result we all are voluntary to take part in the screening process, similarly until they understand the device and its benefit they may ignore it .30, after we create awareness they will accept and use it ,”

Probe: Barriers; problems and concerns about the device

A 23 years old woman said that,” I think there is no barrier on the utilization of this device .but, I double the program mother’s may on table to undergo such screening procedure,”

A 26 years old woman said that,” the other thing is that women my fear and feel embarrassed to expose themselves to there. as result o this they may prefer not be screened and they may prefer traditional halting activities like going to holy water,

A28 years old woman said that `The major barrier is lack of knowledge about the device and its use .If all community member understand this conditional the device there will not be any concerns.`

A25 years old woman said that` the issue of cervical concern in view to our community members. So they may fear to expose the gentle organ for soaring purpose only without feeling apian.

Probe: What do women consider as barrier not to use the device?

A 27 year's old woman said that, `some woman may fear the device. Because then think if that the device may harm their internal body parts. But if we tell about the device and as it have no side effect they will use it.

A 26 years old woman said that, `Other woman fear whether they have the problem or not,. The start to be concerned about the treatment option or service for the problem .This lack of appropriate treatment service in our area may create fear or may enable them not to undergo the screening .`

A26 year’s old woman said that` As said above people fear the treatment charge they will pay if they have a problem. For example .I also concerned about the amount I pay for treatment if I have a problem. `

A28 year’s old woman said that` Husband may not give permission to be screened by looking at the device. He may think that the device may prom bite sexual inter course. This comes from lack of knowledge .So Avery one will use it after they know it appropriately .Additional to this the treatment cost if the problem exist is another issue that create fear on the woman.

A26 years old woman said that ,`woman may feel embarrassed exposing their genital to health professional If it will be done at aver all woman will use it this was a near history on delivery service.

Question 10:- How do you think our self sampling project will be possible in your community?

A24 years old woman said that ` For woman like me who comes from rural areas,1 prefer if the screening will be done at health post level If it is at home I may not able to bring back the device on time to health center .I may also forget it .There are a lot of children in my home .They may

take and play with it when I am not at home .If it is at health center immediately after taking the sample we will give for health extension worker there.

A26 years old woman said that` If it is at home we may say I will take it tomorrow .When we become busy by our daily life activities we may forget to take the sample .As result if possible ,it will be best if it was done at health post level.

A26 years old woman said that `I prefer health center for self sampling process .We all have different daily life activities .During such condition we may forget to take the sample It will best if it is done at health center.

A25 years old woman ` I prefer it may home for self sampling procedure If it was at home every one is not expected to come at health center .This increase the participation of woman in the community .especially those who are unable to come to health center.A24 years old woman said that ` In my option It should be done at home level. I cannot forget my health issue like other materials. I will take the sample appropriately and then I will bring it to health professionals`

A 23 year's old woman said that ` We may make many defaults on sampling procedure like touching the upper surface .To be effective it is better to do screening at health center.

FGD-3 Transcription

FGD for Health Development Army for the perception of Cervical Cancer screening and self-sampling

Socio-demographic characteristics of the participants

Participants code	Age Range	Educational status
1	30-35	10+3
2	30-35	10+3
3	30-35	9
4	30-35	12
5	25-30	10
6	20-25	10
7	25-30	10+2
8	25-30	10
9	25-30	9

Interview: what is Cervical Cancer?

P1: When there is a wound at the crest of the cubical and there is fluid at the cervical

Probe: What are the signs and symptoms of cervical cancer?

P1: It needs to itch that part in addition to fluid at the cervical

Probe: what are the causes of cervical cancer?

P1: There are different causes like unsafe sex, through sexual transmitted diseases and when our body exposed to sun light after waiting in wet environment which is sunburn and is Amharic called “mechi”.

P2: There is high sense of itching in the end part of the cervical and caused by sexual transmitted diseases and when giving birth traditionally

P3: It is a bandit in our cervical and there is liquid

P4: It needs itching and liquid thing in the Cervical and caused by STD and in Amaharic language called “Gerefta”

Probe: What is Gerefta?

P4: this is a condition when we piss in the place of sunburn, it will be evaporated to our body then the diseases will be caused in this condition and we have to be treated immediately.

P5: The causes are early marriage, sitting in the sunburn stone and gerefta and the signs are itching , liquid and wound in the area

P6: usually our community believe that caused by gerefta, early marriage and the signs include disurea during piss even difficult to move in walking.

P7: I think the main cause is gerefta (sun exposure) and mainly the commercial sex- workers are the risk groups because of multi sexual partners.

P8: after raining if sunlight is there, there will be evaporation and during this time if we piss we will exposed for the disease and also early marriage is another cause. The signs are yellow fluid from our cervical and itching.

P9: early marriage, home and traditional delivery are the main cause for the disease and the symptoms are frequent piss ,wound and itching of the area .

Interview: what is your experience , is there any one you know who are exposed for cervical cancer?

P1: yes, I have a sister who is sick in this disease and the signs and symptoms were typical of cervical cancer. She is currently getting the treatment from Gonder hospital.

Probe: what is the perception of the community for these persons who are diseased with cervical cancer?

P1: The disease consequence like bad smelling. So that there is stigma from the community like fistula. currently there is some improvement in the currently perception because of awareness creation

Probe: What seems awareness creation among the community for this disease?
P1: we are the leaders at women and sometime we provide this issue as agenda to the women, so that there is some improvement

P2: I have a friend who was exposed for this type of diseases which show typical signs and symptoms of this disease. She was saved herself from screening because of a shamed for a long time and once a day she told me her sickness then we went

together to Gonder hospital and she was given a drug. Currently she is in good and healthy.

Probe:- what is the perception of the community for persons sicked in this type of disease?

P2. The communities have little awareness for this diseases and it will be severe because of not given treatment immediately.

P3: yes, then was my neighbor who exposed for this disease. She was late to go to treatment especially to Gondar hospital. She was treated many times in the local clinics but not getting improved then when she go to Gondar hospital it was sever and getting operation .now she is improved but not completely healthy. Previously people considered the disease as untreatable but after this women, the perception is improved among the community even to be screened.

P4: Currently persons are getting different health information from different sources like media (TV). so that they understand that what cervical cancer is. Previously it was called generally as cancer and the prevention was not known.

P5: I have no real experience in knowing such patients but generally the community is improved the perception for this disease.

P6: even if my case is different, there were some symptoms and I openly talk to my neighbor and they said that this is because of sunburn (gerefta) and I went to health center and get treatment.

Probe: what is the perception of the community?

P6: currently there is media like (TV and radio) and people are becoming know about the diseases and the perception is good. Even the community share and contribute money for such poor patients to get them treatment.

P7: currently there is no stigma among the community to patients with this disease and there is also awareness creation using one to five network of our social mobilization strategies .

Interview: what seems the cervical cancer screaming service in your area?

P1: the screening service is not started in our district which was not given so far.

Probe: what is your experience in using the service?

P1: We are leaders of one to five networks of the women, so that in any service firstly we used to be role model for others

P2: Women get the service out of this district like Gondar hospital and Debark hospital

P3: before this time the service was no given here in our district and the community also had no awareness to be screened but now the awareness of the community is improved and also getting the service from Gondar and Debark hospital.

Interview: what are the barriers to get the service?

P4: the service does not exist here in our district health center, so that even If we want to get the service but we could not.

Probe: what about is the perception of the community regarding to the service?

P4: Previously women used traditional medicine which is not known its consequence but not the knowledge of the women is improved and getting the service .

Probe: what are the barriers to get the service in your district?

P4: Having shame is the first barriers among the women to get the service. This is because of sensitive issue which is related to increase their awareness.

P5: One of the barriers is being ashamed among the women which should be improved and women get the service after the disease is very sever and threat because of they save from getting the service. Another barriers is the service is not exist here so that women need to go other places which also request money for transport and other services and it makes them difficult to go to the service .

P6. The service is not given here in the health center even when I myself observe some signs like blow fuse when piss, the nurses do not recommend to laboratory service simply they related it to having pregnancy and they give us some drugs which may not appropriate.

Probe: what about the perception of the community to get the service?

P6: the awareness is very low and being shameful. For myself when I see some symptoms I preferred to use some traditional medicine than to get the service because I am shame to speak for the nurses or physicians.

P7: the service is not totally given in our district. Actually we did not know even its name specifically as cervical cancer before we know as the general name as cancer.

P9: the screening service is not here and I myself even did not get the service even if I wanted.

P10: For myself I have seen some symptoms from myself but even if I wanted to get the service I could not get. so that I plan to go other places.

Interview: As you have observed the procedure about the new instrument (self-sampling) from the physician, how it is your perception to use it at individual level in the living house?

P1: This is very interesting equipment and will be best if it is distributed at health post level to use by the community health workers and also good for the women to avoid shame. But I perceive that it will be difficult to use at house hold level especially in the rural area because of highly exposed to the hygiene problems.

P2: It is good instrument and we are happy to use specially at health post level. Even it is good for these women who suspect the case is good opportunity to use it in their house.

P3: It is good to use at house personally to avoid shame because of it is sensitive issue.

Probe: Who may be support her if she use it in her house from her family member?

P3: It is easy to use which is not difficult to use it at house level.

Probe: what might be the barrier to use the instrument at house level?

P3: There is no problem to use each woman in their house but they should be well trained in its procedure and keeping hygiene. It is better it the service of the equipment is at health institution level to keep its standard.

P5: This equipment is more acceptable if at health institution and as a leader we have to role model to be screened first. If it is at house they may not use it with different perception and reason.

Probe: What will be the main barrier to use the instrument?

P5: having shame will be as barrier in both house and health institution and we have to increase their awareness.

P6: the community will be benefited at health post because of to be home accessible for the community. At house level for myself I will not use it and others specially rural women may not well understand the procedure of the instrument.

P7: It will be effective if it is at health post level and we have to be role model for others. As barrier may be they perceive that it is unknown thing and it may touch our sensitive area of reproductive organ, so that save themselves from using it. Previously women who used Norplant for family planning thinking that as the Cause of the disease when they sick. So that they may consider like this.

P8: I am happy for this instrument because many women were shamed to be screened and I can be used at house level because it is life. actually we need to train them how to use it and better to use at this level. The barrier might be when we observe at the starting time it seems hard but it is soft to use, so women may perceive that difficult thing if they did not have well information.

P9: this is good opportunity for women and better at health post level but if it is at house (individual) level, women may be careless and we need to encourage women to use it in peer to peer education using our network.

Probe: what might be the barrier?

P9: women may consider it as hard and suspect to touch sensitive area of our reproductive organ, so that we need to increase their awareness.

Probe: what might be the perception of the husband?

P9: He support me (encourage me to use it, because this is life)

P10: I am happy for this instrument and good either at health post or health center level but not well at house level because there might be hygiene problem which will create infection

Probe: what might be the husband perception?

P10: he might not understand at the beginning because he may perceive that it forbidden sexual relation but if I well communicated him, he will be positive

FGD-4 Transcription

FGD for Nurses for the perception of Cervical Cancer screening and self-sampling

Socio-demographic characteristics of the participants

Participants code	Age Range	Educational status
1	30-35	10+3
2	30-35	10+4
3	35-40	10+4
4	35-40	10+4
5	25-30	10+4
6	25-30	10+4
7	35-40	12+2

Interview: what is cervical cancer and what are the causes?

P2: This is the disease which is usually seen in women in the reproductive age groups and its cause is unknown, the symptoms include bleeding, pain during sexual intercourse.

P3: there is pain, bleeding might be there as the symptoms and the causes are unknown.

P4: as number 3 said the risk groups are the reproductive age group and the signs include abdominal pain, disurea when piss, high fluid , bad smelling and the cause is unknown.

Probe: what is the community perception?

P4: they might be perceive that the cause relating with traditional believes

P5: the main cause is human papiloma virus and the risk groups are females who start sex early, women having multisexual partners. Scientifically women who are 49 years and early ages are difficult to be screened.

Probe: what is its sign and symptom ?

P5: lower abdominal pain, bleeding and pain during sex.

P6: it remained as asymptomatic for ages 15-20 and when the sign starts, there will be bleeding, offensive discharge and might be uterus prolapsed.

Interview: what is your experience for women with this case and what is the perception of the community?

P1. I have no actual experience and what I know that women usually late to came have and the disease will be sever and different to treatment.

P2. The reason a woman is not coming is because of shame. Actually it is improved because of increasing awareness

P3. This is because of lack of awareness, women do not came here and late to be screened and they do not know the disease is treatable in the early stage.

P4: my experience is I heard that a women divorce because of protected sexual intercourse, her husband and their neighbor perceive that it might be related to this but she was not screened.

P5. I have no direct experience but generally there is low level of awareness among the community and they related to relational behaviors

P6. People do not consider as it is treatable, they shamed and keep the disease up to severe, then after it consequence divorce and I have no direct experience.

P7: they keep the disease as a secret because of they are threat in divorce by their husband, so that it will be sever and arrive the stage of not treatable. I have observed those cases recently when I train in the Gondar university hospital and touches my heart by their condition. One of them divorce four times without treatment and now arrive untreatable stage and waiting her death. the physician asked her why late to came and she replied that fear of her mother in laws, might ours, relatives from abuse me and finally she was came with help of there. So the awareness should be created among the community.

P8. They came to the health center late when it is severe and the main reason for being late is not to speak for other persons because e of shame.

Interview: what is the service availability in your district/health center?

P1. The service is not given on this health center

Probe: why it is not given and what is the barrier?

P1. No equipment and no sufficient trained nurse

P2. Not only lack of equipment, but also need its own room. I have not seen also the need from the community who came to get this service

Probe: what will be the demand from the community if it funded with the equipment and trained profession?

P2: women will come for the screening service because awareness creation can be given at church or other places with health extension workers.

P3: from our previous experience women will come to get the service if the information is disseminated for the community because in the past time for fistula women easily came when they were informed.

P4: If the instrument and trained profession is there and awareness creating women will come to the health institution to be screened from our previous experience for others services. So that social mobilization is needed after availing the materials and the trained professional

P5: people will come, I am sure. I trained recently and it does not ask sophisticated material. What I trained is to do screening service with that of easily accessible materials called in Amharic ''Acheto''. the health center is ready to fulfill the class and material.

Probe: what will be the barriers?

P5. May be the motivation of the professionals, we should be motivated to save the lives of the mother and the management of the health center also should be motivated to give the service. this is not only the responsibility of the trained nurses rather all professionals. We have to council them, usually we simple diagnose and prescribe drug, this is not enough and we have to mainstream this service.

Interview: based on the procedure of this self sampling instrument what you have observed from the physician what is your perception to use it at private house level?

P8. In my opinion better to do at health center because even if we teach them, they may not care and the instrument is also to enter to services which ask care.

Probe: what about your opinion for women who cannot get the opportunity to came to the health institution?

P8: for distance and rural area may be better at health post level or at house hold level with the help of community health workers.

Probe: what might be your threat to use at house of individuals?

P8: because of hygiene problem, a woman may develop infection and may be exposed for other things.

P1: if the health extension workers trained, they can do for women during their house to house visit time because of distance the women may not benefited.

P2: the barrier might be stigma after screening with self sampling but it is good to do at house level. Awareness creation should be the first step to utilize the service effectively. Previously there were similar for family planning and other services but improved after awareness creation which is similar to this issue.

P3. At this level it is not good to do at individual house level and their husband may perceive that in the other way and give other meaning for those women to screen them with self-sampling. So that first it should be at health post and health center level then good to go to the household level. In the past similar challenges were for long acting family planning methods with given different meaning.

P4: up to the community bring sufficient awareness about the material; it should be started in the health post and health center level.

P5: this self sampling is good to be at house level but my threat is her husband also should be informed with showing the complication ad consequence of the disease. otherwise he may be one barrier. Mother in laws also should be informed because

these are another influential bodies and the service should be started at health post and health centers level.

P6: It is better to start at health post and health center and her husband may oppose that if he is not informed. As barrier there may be shortage of sampling instrument after mobilizing the community.

Key Informant Interview Transcription-1

Interviewer: S. H.

Participant: Husband

Age Range: 45-50 years old

Educational Status: Grade 10

Starting Time: 2:40 AM

Ending Time: 4:05 AM

Interview Setting and process: I got my respondent at Dabat health center. He was 49 years old. We sit together in the health center to start discussion. After the overall information was given, the participant was asked his willingness to participate in the discussion as well as for tape recording. He agrees on participation and tape recording after argument to delete it immediately after transcription. During interview I have used interview guide, note book and tape record after argument to delete after transcription.

Theme 1: Knowledge and perception on cervical cancer

Q1. Knowledge and perception on cervical cancer

Comical cancer is a problem that seems like fistula and change the position of uterus. This will influence the prenames and delivery process among women. I hear this from Health professionals around us, in coordination with uoG. Serialized hospital there is a center to search and treat people with the problem like fistula. Fistula running committer.

Probe. Do you think cc is similar with fistula?

The difference is that cc is caused by sexual transmitted diseases .the other hand fistula a problem may be caused natural problems. That influence the uterus .

Cervical cancer is a health problem caused by different sexual transmitted diseases. On the other hand fistula is on uterus problem cause by nature among women's. However, cervical problems are temporary problem caused by infectious disease.

Q2. Anyone who experience the problem in your community.

Such sexual organ problems will not be open for discussion in our community. The maximum level if knowledge about the problem is at house hold level. So it is limited at that level. I have one sister who faces such problem. During she undergoes sexual intercourse. The uterus will come out to the outside through vagina. But, I have heard some information on sign & symptoms

of cc among community members. So there are many people with such [problem in our community. In collaboration with local health bureou Gondar University hospital will have a screaming program for such women. After they identify the exact problem the will refer to the respective health institutions.

Q3 Haw is the perception of the community on someone with cc?

First of all for women with cc sign & symptom it is difficult to go in to marriage relation ship. They have low chance to engage in marriage rin ship. For example if a her husband smell four smell when he sleep with her he hate to be with her again, on the other hand I f this problem happened after she all ready entered into the marriage, it may end with separation, In summary the sign & symptoms like four smell will disengage the cuamen from the entire social relationships. As a result the will feel isolated from the social segment and activities.

Probe? If this problem influences the marriage, what is the fate this family? If depends on the perception of both the husband of his wife. If he have positive attitude he will try what he can to seek treatment for her problem. On the other hand some who will not be comfortable with the problem? Immidiatry may decide to stop the marriage reship. B/c they perceive as the problem may not have a treatment. So they don’t want to continue with the problem. Finally the will stop marriage rim ship and women will face al lot of social of psychological problems.

Q4 causes of CC

In our local tradition there is a cause factor called “ graffiti” which is caused when women urinate on hot land or when she sit on hot stone or when she exposed he body suddenly to sun light. In our culture this will cause cc. In scientific way it may be caused by disease causing agents that can be transmitted from one to the other. However, “graffiti “ is well known and accepted cause of cc. especially during urination on hot land, the steam from the land directly enter into her body and results this problem,

Q5 cc Screening Service.

The will known Screening Service in our local health facilities was for fistula problem. Other mothers with problem in their uterus will be treated as other health problem in the health center. If the problem is fistula she will directly refer to Gander University fistula clinic for treatment. However, cc, will be screened as other health problems in the health center. If it was beyond the health professionals in the health center they will refer them to the other hospitals.

Q6 what looks the experience with cc Screening?

There are women who say that they have Screened and got treatment for

Different uterus problem including cc.

Q7 what make them to take the screening service?

Women will go for screening service as a result of pressure from the famine members including husbands. Most of such problems are hidden & will not be told to any other person. If they get confidential health professionals they will tell their problem and get treatment for it, But, when health center is busy with people such patent may go back to their home.

Q8 what are the barriers for others not to be screened.

Some wirner will not want to phrase their problem to others, If they disclose this issues to other will result to be challenged by different social pressures for this reason they will keep secret. They will tell this even for their family members. I think there are a lot of women. With this problem who will not tell their problem & will not get. Appropriate treatments. This is bad culture in our community. They will go to treatment when forced by the situations, for example severe pain during inter course.

Probe? What are other potential factors that can be a barrier for cc screening? Some people fear examination and treatment fee of the problem, some problems may be treated at local level. But if it is beyond that, they will fear the causes of referrals and sometime cast for medication. On the other hand advice from their neighbors (if there) also can be a barrier. If they advised to stay at home they will act accordingly.

Q9 who do they prefer to do examination? Why?

Self- examination instruments are not well known and practiced in our local situation, women may not have appropriate skill & knowledge to do so, they will be satisfied if the ge the scarpering service at health institutions, on the other hand most women will have self examination experience before for any health problems, so, it became new for them. However, getting treatment at health institution is well known practice and it will be simple for them.

Probe? How about if we give them brief explanation for them?

As I have said before Confidentiality is the major thing for most women on sexual and reproductive health issues. The major gap for self-examination is lack of Knowledge and experience to undergo the procedure. If they got brief explanation and understand the procedure every women will perform it at their private place.

Probe? Is there a private place for a woman to do so at home? Defiantly the will have a private place. They can arrange the situation at home for having private time to undergo the procedure, for example when any one is not present at home .otherwise , she will have at of options like in bath room, in toilet or in bod room, I think it will not be difficult for women to arrange private place to undergo such screening process,

Thems3 Question on self sampling device?

Q10 what do women's feel about this procedures of the device? I think the procedure is simple and after clear instruction women can do it at their home. This will keep their privacy and Secret which they want. So they will have a good interest to use it if they have hint on the procedure.

Probe? What is required from us on this regard?

It is expected to raise the awareness of the women with regard to the device and how to use it , It will be better if the information giving process on the device is one to one. Or face to face process. But if it is in mass women's may fear to do so.

Probe? Would the need any help in self sampling? If there from whom?

Most of the time women will communicate secret issue to other women of similar age. Additional to this they may tell to their girls. Sometimes she may tell her Condition to her daughter. Her daughter may help her in the process, but, boy children's or husbands may not be requested for help by the women,

Probe? What might be a barrier, problem or concerns not to use the device?

This procedure is a secret that women limit at their own level. If it will done in this way there will not be any barrier. However, husband should be well informed about the device If not they may have another feeling about the device, The will not look it positively like she used it to screen her health condition to keep her health status, But, the time she used to take the sample is, not more that five minutes. This is very short and can minimize any conceras from any body. After she collects the sample she can immediately send it the health processional. So in may opinion nothing will be a barrier for this procedure of device.

Probe? What other conditions need to be considered?

Some women my fear to use the device which results from lack of knowledge about the device. Flow ever; if they understand the prons and cons of the device no one will refuse the procedure. She will do everything for herself for her health condition. If other people perform this procedure for them they may develop certain fear. However, this is self sampling procedures which enable them to keep their secret for themselves. So, there is nothing that will be considered as barrier for this self-sampling procedure. I think self-sampling procedure enhance the interest of the women to use the device.

Probe? Where do women prefer to undergo the procedure? At health center or health post? By whom?

As I have said, If women will not understand about the device, they will not accept the procedure. To do so, health education information should be distributed to the general population at different level. Starting from health center throughout the kebele busing d/t methods. After this

it will be simple for many women's to use the device & the procedure we can learn this from fistula case. Which was hidden for long period of time, after progressive IEC/BCC work now peoples became familiar for it, when the device enter into vaginal they may fear it will harm their internal body, They may think it may harm their utures. If we tell that the device will not harm the utures the will easily use it.

Q11 how do this self-sampling project could be feasible in the community. If it will started at health center if will be good. However, if it is at home people will not give weight/place for the device. Additionally, at home people may touch the upper part with their hand or may not keep it in appropriate place; they may not also appropriately undergo the procedure. At health centre health professionals may assist the women in taking the sample.

Probe? How about at health post?

People have low trust on health extension workers. As a result they may not be voluntary to expose themselves their body in front of them, for example a few times ago peoples were not voluntary to take even immunization by health extension workers, After repeated exposure now they will use the service from them.

Key Informant Interview Transcription-2

Interviewer: S. H.

Participant: Health Center Official

Age Range: 30-35 years old

Educational Status: Public Health Officer (BSC)

Starting Time: 1:40 pm

Ending Time: 3:05 AM

Interview Setting and process: I got my respondent at Dabat health center. He was 30 years old. We sit together in the health center to start discussion. After the overall information was given, the participant was asked his willingness to participate in the discussion as well as for tape recording. He agrees on participation and tape recording after argument to delete it immediately after transcription. During interview I have used interview guide, note book and tape record after argument to delete after transcription.

Theme 1: Knowledge and perception on cervical cancer

Q1. Knowledge and perception on cervical cancer

Most women consider their body below the hub as a futures. The pain at this body port is considered as normal problem of theaters. The did not distinguish cervical cancer from other health problem on & inside reproductive organ, they will not suspect cervical cancer problem. Even if it occurs they generalize as sexual transmitted diseases. However, if we see the sign and symptoms of cervical cancer on patients we will refer to debark hospital for further investigation. But among the community member the issue of cervical cancer is not well known to pic, even among the professional those who trained only have a good Knowledge about the issue and related things. The untrained may not have a good wariness about cervical cancer. So there is high lack of knowledge on cervical cancer among our community members,

probe? What are activities your are doing to enhance this awareness level?

After some professionals trained on Cervical cancer examination /screening we made them to explain the sign & symptom of cc at different community meeting, We also have d/t conferences with HAD (Health Development army) and community members to transmit information on

cervical cancer, these conferences are already utilized for other services specially for maternal and child health issues, we use this program to transfer messages about cervical cervical cancer, However we have not introduced specific sessions for cervical concer issue only. Addition when different health parse ssional come and tell us the problem of this cervical cancer we will made them to transfer their message to the community,

Q2. What are the causes of cc for the commodity? What does the community beliefs about the cause of cc?

The community member will of identify the problem and its sign and symptoms. There are different problem related to reproductive health among women including prelease, during such situation the community will go to traditional conditions like holy water. After the problem become complicated the will come to health institutions, then we will refer them to university of Gondar hospital for treatment. For example we this year we have identified to cases of uterus prolabse with the help of health development army and referred them to university of Gondar hospital.

Probe? How about the causes? I think the community may have its own beliefs regarding the causes of cc. I don’s know most of them. But, we can identify these beliefs by asking the community members. Some people think it is god’s punishment.

Q3 How is the perception of the community. On someone with cc?

Most of people who life in this area was from rural part. If they see anything that deviate new from normal physiology. They will have different attitude and feeling, those with the problem may feel,

Embarrassed and may not come to health institution early, this problem may also affect marriage relationship. The husband may blame her for the cause of the problem or she may blame him for the problem, they may develop mistrust on each other, on the other hand such problems like cc require introducing storing rapport with the patient, If not they will not tell the exact sign & symptom of the disease,

The sign & symptom of cc is difficult to tell to other, for example, severe point during intercourse, when I was a clinical staff at health center I have seen different women who say I

am unable to have health sexual inter course with my husband, the feel strong point during sex. This may result in separation of the marriage r/n ship. Additional to this foul smell discharge may offence husbands, as a result their husband prefers not to be with them this have serves social impact.

Theme—2 Questions on cc screening

Q-5 : cc screening opportunity in the district.

On cc screening two health professional were trained from our health center, they were two midwifes trained on dopier and examination of cervical cancer screening, other than this we recommend the women to go to debark hospital for screening. Some people may get Gander university hospitals for screening. Even if, there are trained midwifes on cc screening, we will not started the screening service until now. For women with certain sign and symptoms that looks cc we way refer to the hospital for ultrasound checkup, so currently we will not do cc screening in our health center, as a result most of the community , member are unable to under go cc screening,

Probe? What is the experience of the people (community) with cc screening?

First of all in our health center we will have an equipment as well as man power to under go the screening process. So, the service was not available and accessible to the community member. If the service is available to the community and if we work on enhancing the awareness on cervical concern they will use the screening service.

Question 6 what are barriers for them not to be screened?

Women feel embarked to be examined (screened) for cervical concern. Even among health professionals there is a strong fear and & mabarasmeny to be sceered for cc through vaginal examination. This is because of traditional beliefs among the community members, to avoid this fear and embracement we need to work on enhancing the ware mess of the community the other barriet is availability of the service at the near by health facilities.

Question8? Who do they prefer to do the examination? Where?

Self sampling process of Screening is good to avoid fear among women, If will be very conformable for many women

Probe? What will be a barrier for self sampling process?

To avoid potential barriers an self-sampling process we need to counsel women with their partner. This will prevent husband permission not to be a barrier for bereaving process, additional to this she can arrange private place to take the sample.

Question 9 what do peoples feel about this procedure of the device?

As I have seen if, the device is simple, and good which can be used by every women if we, tell them about it, If is not difficult. And every woman can under go self-sampling procedure at home,

Probe? What type of instruction will be needed for each women?

First of all we should discuss the issue by sitting to get her with all women. This should be clear discussion introduction between groups to under stand the instrument of its benefit,

Probe: Who would they need to help in self sampling? This is very simple procedure; any women can do it with out any support from other individuals.

Probe: what might be barrier and concerns on this device?

Women may not give attention to word self- sampling procedure. But if we clearly educate (give information) about the severity of cervical concern there is no way that the women will ignore it, then they can easily use it.

When we start our work we may identify the parietal problems But currently I will not consider any thing as a barrier for this device and procedure. As we don’t start the screening procedure we will not know the exact things that can be a barrier for it,

Question 10 how do you think self-sampling project could be effective in the community?

To be effective we can use health extension workers and health development army. So, by working with them at each kebeles we can enhance the effectiveness of our project. We will

educate them, and they will also transfer that information to women's under their leader ship, so the best way is through HAD's and health extension worker,

Probe? Where should be the best place of self-Sampling?

We can use it at individual's level or we can also arrange one class at health center for Screening purpose, to collect the sample correctly and with out contamination it is best to collect it at health center under the support of heath professionals. But if we show them appropriately we can we self-sampling procedures at individual level.

Probe? Do women prefer make or female professionals to support them in self-sampling?

I think they may prefer women health professionals to under go self-sampling. Be cause women may develop fear and embarrass many in front of male professionals, so, it will be best if women health professional do the screening.

Key informant interview 3 with midwife

Interviewer: G. D.

Participant: Midwife

Age Range: 30-35 years old

Interview: what is Cervical Cancer?

Response: it is a disease caused by human papilloma virus. Early sexual intercourse is as one cause and being commercial sex workers who does not use condom are more risky.

Interview: what is your experience, is there any one you know who are exposed for cervical cancer?

Response: yes, I have experience. There was a women who is my near relative dies with this case by extremely bleeding and with bad odor liquid

Interview: What are the signs and symptoms?

Response: there was pain in her lower abdominal part, bad liquid odor and lower appetite

Probe: Did not she get treatment?

Response: she went to Gondar hospital but it was irreversible stage and told by her doctor, she wait simply her death

Interview: what is the perception of the community for these persons who are diseased with cervical cancer?

Response: the disease is assumed very sever, there is liquid and bad odor so that except the family they do not want to approach other people. There is stigma among them, the community believes that it is untreatable disease and called them in Amharic language “nekersa”.

Interview: what seems the cervical cancer screaming service in your area?

Response: we have trained in screening service in our institution. I personally request the institution/ the health center (speculum and acetic acid) but could not provide us. So that we are not serving the community. It was better the organization who trained us to provide the necessary equipment.

Interview: what about the perception of the community to get the service?

Response: currently we did not get perception specifically for this service but I understand that if the community well aware about the disease they will have interest to get this service

Interview: what will be the barriers to get the service in your district?

Response: for me the main challenge will be having independent room for this service because there is no sufficient rooms in this health center because the service needs high confidentiality

Probe: what will be the barriers from the community side to get the service?

Response: there may be shame to not to expose their organ/ vagina to providers because we are observing from the delivery experience. Mothers

may assume that this service may be given simply without diagnosis but if they are aware they will be improved

Interview: what seems the creation awareness session for this disease at health center and district level

Response: there is no previous experience at both level, actually for our level we want to create awareness for mothers who came for other services but as I have told you without fulfilling the instrument what is its importance to give awareness? What we do if they came? So this is the reason that not to focus awareness session

Interview: As you have observed the procedure about the new screening instrument (self-sampling) from the physician, how it is your perception to use it at individual level in the living house and what will be the possible barriers at house level?

Response: I hope it is nice instrument because it prevents the shame of mothers to not to expose their organ/vagina for the providers but it needs repeatedly giving instruction and also sufficient aware for husbands because as mentioned above he may understand mistakenly

Interview: whom family member do you think that best to help her if the mother want?

Response: I think her husband is best by aware him because some husband may think mistakenly

Interview: which level is most effective to use self-sampling at house individual level, health post or health center level?

Response: of course individual level is best because of the above reasons but at health post level is also will be effective because of health extension workers are very intimate relation with mothers and possible to do it and health extension workers then will bring the sample to the health center.

Key informant interview 4 with Health extension worker

Interviewer: G. D.

Participant: Community Health Extension Worker

Age Range: 30-35 years old

Interview: what is Cervical Cancer and its cause and symptoms?

Response: its symptoms include itching, bad odor etc

Interview: what is the community perception and awareness?

Response: no one has awareness for this disease

Interview: what seems the cervical cancer screening service in your area?

Response: the service is not given in our area but I have information that it is given in University of Gondar specialized hospital

Interview: what will be the barriers to get the service in your district?

Response: mothers may hide the issue. Usually our community do not discuss freely this type of issue but if we give sufficient awareness the community will have interest to get this service

Interview: As you have observed the procedure about the new screening instrument (self-sampling) from the physician, how it is your perception at which level will be effective? At individual level in the living house, at health post level or at health center level?

Response: for the urban community it will be good if it is at health center level and for the rural community it is possible to give at health post level but at house level there might be hygiene and sanitation problem for the instrument which may cause infection for mothers/women

Interview: whom family member do you think that best to help her if she want at house level and preference of professionals at health center?

Response: mothers preferred female child at house level if she want help and female nurses and midwives are more preferable at health institution level for mothers screening from previous experience
